# Sex and performance‐level differences in aerobic and haematological parameters among elite ski mountaineering athletes

**DOI:** 10.1113/EP093131

**Published:** 2026-01-23

**Authors:** Forrest Schorderet, Bastien Krumm, Basile Moreillon, Justin Mottet, Antoine Raberin, Nicolas Bourdillon, Raphael Faiss, Grégoire P. Millet

**Affiliations:** ^1^ Institute of Sport Sciences University of Lausanne Lausanne Switzerland

**Keywords:** aerobic capacity, haemoglobin mass, sex difference

## Abstract

Ski mountaineering (SkiMo) is a new Olympic sport with extreme endurance demands and altitude exposure. Previous studies have focused on traditional cardiorespiratory variables, such as maximal oxygen consumption (${\dot{V}}_{O_{2}\max}$) or ventilatory thresholds, but, to our knowledge, did not report haemoglobin mass (Hbmass). The aim of this study was to investigate the relationship between Hbmass and ${\dot{V}}_{O_{2}\max}$ in elite SkiMo athletes and compare physiological differences across performance levels. Twenty‐nine Swiss national team SkiMo athletes (10 females and 19 males) were classified into Tier 3–5. Participants performed a treadmill graded exercise test (25% slope) to determine ${\dot{V}}_{O_{2}\max}$ and ventilatory thresholds. The Hbmass and blood volumes were assessed using a CO‐rebreathing technique. Sex and tier‐based comparisons were made, and correlations between haematological parameters and aerobic performance metrics were analysed. The Hbmass normalized to body mass (HbmassBM) was significantly correlated with ${\dot{V}}_{O_{2}\max}$ in the pooled group (*r* = 0.80, *P* < 0.001), females (*r* = 0.82, *P* = 0.007) and males (*r* = 0.53, *P* = 0.024). The Hbmass and related haematological parameters were significantly higher in males (*P* < 0.05). Males in Tier 5 had higher oxygen consumption at the second ventilatory threshold (63.0 ± 4.3 vs. 58.9 ± 2.8 mL min^−1^ kg^−1^; *P* = 0.022) and ${\dot{V}}_{O_{2}\max}$ (72.0 ± 4.4 vs. 67.4 ± 3.1 mL min^−1^ kg^−1^; *P* = 0.015) than those in Tier 3–4. The significant correlation between HbmassBM and ${\dot{V}}_{O_{2}\max}$ confirms the key role of Hbmass in oxygen transport and aerobic capacity. However, Tier 5 athletes achieved superior aerobic performance without higher HbmassBM, indicating that additional physiological factors underpin elite‐level performance.

## Abstract (highlights)

- **What is the central question of this study?**
  What is the relationship between haemoglobin mass and maximal oxygen consumption or performance metrics in male and female elite ski mountaineers? Data were previously lacking on athletes who train and compete at altitude. We questioned whether haemoglobin mass is higher in this population compared with other elite endurance athletes.
- **What is the main finding and its importance?**
  Values of haemoglobin mass in ski mountaineers were consistent with those in other endurance athletes. Significant correlations between relative haemoglobin mass and maximal oxygen consumption were observed. Overall, this study confirms haemoglobin mass as a key determinant of aerobic performance in ski mountaineers, highlighting its relevance in this altitude‐based sport.

## 1 INTRODUCTION

Ski mountaineering (SkiMo) is a new winter Olympic sport to be featured for the first time at the Milano Cortina 2026 Olympic Games. It requires both exceptional physiological capacity for uphill skiing and technical skills for downhill skiing (Bortolan et al., 2021). A recent study (Fornasiero et al., 2025) showed that higher maximal oxygen consumption (${\dot{V}}_{O_{2}\max}$) and oxygen uptake (${\dot{V}}_{O_{2}}$) at the second ventilatory threshold (VT_2_) were associated with better SkiMo performances. Uphill skiing is the primary determinant of overall performance in sprint and mixed relay events, which are the selected events at the Olympics, and accounts for 80%–90% of the performance variance (Fornasiero et al., 2025). High ${\dot{V}}_{O_{2}\max}$ values have been reported in world‐class SkiMo athletes, with average values of 64 mL min^−1^ kg^−1^ in females and 73 mL min^−1^ kg^−1^ in males (Schorderet et al., 2025). These values are consistent with previously reported data in elite athletes (69.3 ± 7.4 mL min^−1^ kg^−1^ by Fornasiero et al., 2017; 71.2 ± 6.8 mL min^−1^ kg^−1^ by Lasshofer et al., 2021), reinforcing the importance of aerobic power at the elite level.

Given that endurance athletes rely heavily on efficient oxygen transport to working muscles to sustain high aerobic performance (Levine, 2008; Wagner, 2000), understanding the physiological determinants of SkiMo performance becomes essential. Key determinants of ${\dot{V}}_{O_{2}\max}$ include cardiac output and arterial oxygen content, the latter depending largely on total haemoglobin mass (Hbmass) (Schmidt & Prommer, 2010). Although elite endurance athletes have high values of Hbmass (Oberholzer et al., 2024), most also exhibit relatively low haemoglobin concentrations ([Hb]) owing to exercise‐induced haemolysis and plasma volume expansion; a phenomenon known as ‘sports anaemia’ or ‘pseudoanaemia’ (Eichner, 1992). Haemoglobin (Hb) is the oxygen‐carrying protein in red blood cells that binds and transports oxygen throughout the bloodstream (Green et al., 1999; Mercer & Densmore, 2005). A strong association between Hbmass and ${\dot{V}}_{O_{2}\max}$ has been documented, with evidence suggesting that an increase of 1 g in Hbmass can lead to an increase of ∼3–4 mL min^−1^ in ${\dot{V}}_{O_{2}\max}$ (Prommer et al., 2007; Schmidt & Prommer, 2010; Webb et al., 2023). A high Hbmass is, therefore, a defining trait of endurance athletes. Male and female elite endurance athletes typically have Hbmass values of >13.5 g kg^−1^ (Heikura et al., 2018; Heinicke et al., 2004, 2001; Mancera‐Soto et al., 2025; Oberholzer et al., 2024; Wehrlin & Marti, 2006; Wehrlin & Steiner, 2021) and >11.0 g kg^−1^ (Garvican et al., 2010; Heikura et al., 2018; Heinicke et al., 2004; Oberholzer et al., 2024), respectively. As expected, these values are significantly higher than those of untrained individuals (Böning et al., 2004; Heinicke et al., 2001). Sea‐level training has been reported to have minimal direct impact on erythropoiesis, with genetic predisposition suggested as a key factor for high Hbmass and endurance performance (Schmidt & Prommer, 2008).

Although the relationship between Hbmass and ${\dot{V}}_{O_{2}\max}$ or performance has been well documented, most research has focused on summer sports, such as triathlon and cycling (Goodrich et al., 2018), running and rowing (Gore et al., 1997) or swimming (Mujika et al., 2023). However, the relationship between Hbmass and ${\dot{V}}_{O_{2}\max}$ in elite endurance winter sports is comparatively sparse. Moreover, several factors might alter Hbmass values, including training status, sex and iron deficiency (Goodrich et al., 2020). Male athletes typically exhibit greater lean mass and lower body fat percentages (Goodrich et al., 2018). Although Hbmass and lean body mass have been shown to be correlated (Oberholzer et al., 2024; Prommer et al., 2018; Schumacher et al., 2008), the differences in Hbmass between sexes and across sports disciplines are not explained solely by variations in lean mass (Goodrich et al., 2020).

Another important influential factor for Hbmass is hypoxia. Altitude training camps, which are regularly used by summer and winter elite endurance athletes (Millet & Brocherie, 2020), can induce a 2%–9% increase in Hbmass (Hauser et al., 2016; Heinicke et al., 2004; Saugy et al., 2022; Saunders et al., 2013; Wachsmuth et al., 2013). Moreover, supporting the influence of chronic hypoxic exposure on haematological adaptations, young and junior male cyclists born and training at moderate altitudes (≥2500 m) have been reported to exhibit slightly higher relative Hbmass values than their low‐altitude counterparts (Mancera‐Soto et al., 2025). Although ski mountaineers are not always born at moderate altitude, they are regularly exposed to altitude owing to the unique demands of their sport (mountain sport; need to reach the snow on glaciers regularly for specific on‐ski sessions) for both training and competition. Elite ski mountaineers are therefore considered endurance athletes exposed to high hypoxic dose, i.e., the cumulative time spent in a low‐oxygen environment (Faiss et al., 2014). For instance, they can train up to 1000 h per year, with >50% of this time spent above 1500 m, particularly during SkiMo and trail running sessions (Faiss et al., 2014). The hypoxic dose reflects both the altitude and the duration/frequency of exposure and is a major determinant of haematological adaptation (Garvican‐Lewis et al., 2016; Millet et al., 2010; Wehrlin et al., 2016). Of interest, the hypoxic dose is typically expressed either in hours of exposure or as kilometre‐hours (km h) (Garvican‐Lewis et al., 2016), which normalize time to altitude. However, these metrics do not fully capture the complexity of individual physiological responses to hypoxia. Therefore, an alternative approach has been proposed, based on continuous monitoring of pulse oxygen saturation ($S_{pO_{2}}$) to quantify the ‘effective’ hypoxic stimulus better (Millet et al., 2016). The erythropoietic response to hypoxia is influenced by several additional factors, such as the type of exposure (e.g., live high train high (LHTH), live high train low (LHTL), intermittent hypoxic exposure (IHE)), individual acclimatization and marked interindividual variability. For instance, some athletes might exhibit a slight decrease in Hbmass, whereas others show increase up to +10% following an 18 day altitude camp (Hauser et al., 2016).

To our knowledge, there is a lack of data on both male and female elite ski mountaineers. Previously, we investigated aerobic performance parameters during graded exercise in elite SkiMo athletes (Schorderet et al., 2025), and the present study extends this work, on a different group of athletes, by evaluating total Hbmass and blood volume (BV); variables scarcely reported in this population. We aimed to explore potential sex‐related differences in these haematological and physiological characteristics, given the limited evidence available in elite female SkiMo athletes. Therefore, the present study aimed to address existing gaps by examining Hbmass and its relationship with ${\dot{V}}_{O_{2}\max}$ and other performance metrics in elite SkiMo athletes. A strong correlation between Hbmass, aerobic capacity and performance level was expected. Additionally, we hypothesized that SkiMo athletes would exhibit higher Hbmass values than those typically reported in other elite athlete populations. Finally, we expected ${\dot{V}}_{O_{2}\max}$ to differ between performance levels.

## 2 MATERIALS AND METHODS

Twenty‐nine elite SkiMo athletes (10 females and 19 males) from the Swiss national ski‐mountaineering team participated in this study. They were classified into national, elite or world‐class levels (Tiers 3–5; Table 1) based on an established ranking system (McKay et al., 2021). Tier 3 corresponds to national‐level athletes, Tier 4 to elite international competitors and Tier 5 to world‐class performers (Table 1). To improve clarity, athletes were further categorized operationally as follows: Tier 3 included youth‐category athletes (U20) competing nationally or in junior international events; Tier 4 included senior athletes who had participated in World Cup competitions without achieving a podium finish; and Tier 5 included senior athletes who had obtained at least one podium in a World Cup, World Championship or European Championship event. The athletes, aged 18–32 years, had all previously competed in multiple junior or senior SkiMo events at the World Cup level. All athletes available from the Swiss national team during the pre‐season were invited to participate, and no additional selection or random sampling was performed.

**TABLE 1:** Number of athletes classified by Tier and sex.

Tier classification	All	Female	Male
Tier 3 (highly trained/national level)	9	4	5
Tier 4 (elite/international level)	8	2	6
Tier 5 (World Class level)	12	4	8

*Note*: Tier 3, athletes in the Youth category (<20 years old); Tier 4, athletes who have competed in World Cup events but have not finished on the podium; Tier 5, athletes who have secured at least one podium finish in World Cup races, World Championships or European Championships.

Athletes resided at a mean altitude of 906 ± 344 m (range, 360–1550 m). During the year, athletes accumulated a typical ‘hypoxic dose’ through 2–3 weeks of preparation camps on glaciers in November (i.e., skiing at altitudes of >3000 m a.s.l. while sleeping at 1600–1800 m a.s.l.) and by training and competing on weekends at altitudes of >1500 m a.s.l. Among those competing in the senior category, 12 athletes had achieved at least one podium finish in World Cup races, 7 had secured at least one podium at the World Championships and 3 had reached the podium at the European Championships. Of these 29 athletes, 19 had participated in a previous study (Schorderet et al., 2025); however, the present study involved different physiological measures and was conducted in a separate competitive season. Therefore, none of the present results have been reported previously.

Participants provided a written and fully informed consent to volunteer in the study conducted in adherence with the *Declaration of Helsinki*, with risks and benefits of the applied procedures validated by the local ethics committee (CER‐VD, SwissEthics Project #2023‐02262).

### 2.1 Protocol

**FIGURE 1:**
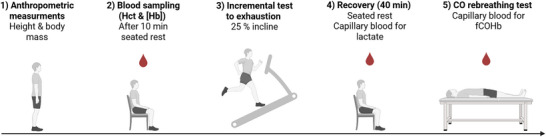
Overview of the experimental protocol. Anthropometric measurements (height, body mass and skinfold thickness at the right vastus lateralis) were performed first. After 10 min seated rest, capillary blood was collected for haematocrit (Hct) and haemoglobin concentration ([Hb]). Participants then completed an incremental treadmill test to exhaustion (25% incline; speed increased by 0.3 km h^−1^ min^−1^; women started at 3.5 km h^−1^, men at 4.0 km h^−1^). During 40 min of seated recovery, capillary blood was sampled for lactate at multiple time points. Finally, a CO rebreathing procedure was performed in the supine position, fraction of carboxyhaemoglobin (fCOHb).

Participants were instructed to refrain from consuming caffeine or alcohol and from engaging in intense exercise for ≥24 h prior to and on the day of testing. Upon arrival, classic anthropometric measurements were recorded, including height, body mass and skinfold thickness at the right vastus lateralis. Following a 10 min seated rest, capillary blood samples were collected for haematocrit (Hct) and [Hb] determination prior to exercise. Participants then performed an incremental treadmill test to exhaustion on a 25% incline. After a 40 min seated recovery, a CO‐rebreathing test was performed in the supine position (Figure 1).

Given the increasing evidence to suggest that exercise performance, including exercise in hypoxic conditions (Citherlet et al., 2024), does not vary significantly across the phases of the menstrual cycle in eumenorrhoeic women (Elliott‐Sale et al., 2020), the protocol did not schedule female athletes according to a specific menstrual cycle phase. However, this information was recorded for ongoing intra‐athlete monitoring.

### 2.2 Anthropometric measurements

Height was measured using a wall‐mounted stadiometer, and body mass was recorded with a digital scale (Trisa 1858, Triengen, Switzerland). Skinfold thickness was assessed on the right vastus lateralis, ∼10 cm above the knee joint, using the Holtain Skinfold Caliper (Holtain Ltd, Crymych, UK), which provides measurements with a precision of 0.2 mm. Each skinfold measurement was repeated three times, and the mean value was calculated.

### 2.3 Performance measurements

The physiological assessment was conducted using a graded exercise test to exhaustion. Briefly, the test was performed on a motorized treadmill (Hp Cosmos T170, Cosmed, Rome, Italy) set at a constant 25% slope, with athletes using trail running poles. Speed was increased by 0.3 km h^−1^ each minute, corresponding to a 75 m h^−1^ increase in vertical velocity (vV). Athletes started walking (females, 3.5 km h^−1^; males, 4.0 km h^−1^) and transitioned to running when needed, continuing until exhaustion while secured with a harness. Finally, participants rested for 40 min before Hbmass and BV determination.

Gas exchange was measured continuously with a calibrated breath‐by‐breath analyser (Quark CPET, Cosmed, Rome, Italy). Heart rate (HR) was recorded using a Polar H10 HR sensor (Polar, Finland), and pulse oxygen saturation ($S_{pO_{2}}$) was monitored at the earlobe with the Nonin 8000Q2 earclip (Nonin Medical Inc., Plymouth, MN, USA).

Lactate levels were measured during recovery at 1, 3, 6, 9, 12, 16, 20, 25 and 30 min postexercise. Blood samples (20 µL) were collected from a finger using and analysed for lactate concentration with the Biosen C‐line Clinic system (EKF Diagnostics, Cardiff, UK).

### 2.4 Determination of circulating blood volumes

The [Hb] and Hct were measured before the graded exercise test to avoid the haemoconcentration effect of intense exercise. Subjects remained seated for 10 min before these measurements were taken. Capillary blood samples were collected from a fingertip. The first drop of blood was discarded, and the second drop was collected into a microcuvette holder for immediate analysis using the HemoCue Hb201+ (HemoCue AB, Ängelholm, Sweden). Two measurements were performed to reduce the analytical variability. Subsequently, Hct measurement was conducted using 40 mm capillary tubes. One tube per subject was filled and placed in a centrifuge (SERVOspin SmartPro, Wesel, Germany) before analysis through the micro‐haematocrit reader.

The Hbmass and BV were determined after the graded exercise test by using a semi‐automated CO‐rebreathing method (OpCo, Detalo Instruments, Denmark). The detailed procedure is described elsewhere (Siebenmann et al., 2017). The CO‐rebreathing procedure was performed in the supine position, in a quiet laboratory room with controlled temperature and humidity. Briefly, participants underwent a 6 min inhalation of a gas mixture containing medical‐grade oxygen (O_2_) and pure CO (99.997%, N47 CO, Carbagas, Gümlingen, Switzerland)), followed by 4 min of breathing ambient air for gas mixing. Administered CO doses were 1.0 mL kg^−1^ for men and 0.8 mL kg^−1^ for women. Capillary blood samples were taken before and after the 10 min procedure, with carboxyhaemoglobin (HbCO) analysed in triplicate with a dedicated blood gas analyser (ABL‐800, Radiometer, Denmark). The remaining CO in the system was measured by a pre‐calibrated CO sensor (Dräger Pac 5500; Drägerwerk AG, Lübeck, Germany) to determine the dose effectively inhaled. All measurements were performed at ∼400 m a.s.l. Participants remained lying supine with their legs elevated throughout the procedure to optimize CO mixing, refrained from speaking and used a heated hand‐warmer to facilitate rapid capillary sampling (three capillaries collected within ∼15 s).

Because haematocrit was determined from fingertip capillary samples rather than venous blood, no additional correction beyond the built‐in algorithms of the Detalo Performance software was applied, consistent with Siebenmann et al. (2017).

We did not prescribe a specific hydration protocol. However, athletes were instructed to follow their habitual optimal hydration routines on test day. Given their elite status and the importance of arriving well hydrated for performance testing, it is reasonable to assume adequate hydration levels.

Finally, based on ΔHbCO, [Hb] and Hct, the Hbmass, total blood volume (BV), red blood cell volume (RBCV) and plasma volume (PV) were determined with formulas described elsewhere (Siebenmann et al., 2017). The typical error of measurement recently reported by our research group with duplicate measurements is 1.6% (Krumm et al., 2024).

One female athlete did not perform the Hbmass test for personal reasons, and one male athlete was unable to perform the Hbmass test owing to technical issues.

### 2.5 Data analysis

Gas exchange data were processed using MATLAB (v.R2023b; The MathWorks, Inc., Natick, MA, USA). The ${\dot{V}}_{O_{2}\max}$ was defined as the highest 20 s average value. First and second ventilatory thresholds (VT_1_ and VT_2_) were identified through visual inspection by two experienced physiologists, as outlined by Beaver et al. (1986). The 20 s average preceding each was calculated for all parameters. These 20 s average ${\dot{V}}_{O_{2}}$ values at VT_1_, VT_2_ and maximal effort were subsequently used to calculate the vertical running economy (REv; in millilitres per kilogram per vertical metre), by dividing the averaged ${\dot{V}}_{O_{2}}$ by the corresponding vV at each intensity level. Regarding the maximal vertical velocity (vV_max_), the velocity of the last completed stage was recorded for each athlete. The maximal blood lactate concentration ([BLa]_max_) was defined as the highest measurement recorded during the recovery phase, because blood lactate typically continues to rise for several minutes after maximal exertion (Gupta et al., 2021).

Male athletes in Tier 3–4 were pooled and compared with Tier 5 athletes. This analysis was not possible in women owing to the smaller sample size.

### 2.6 Statistical analyses

Data are presented as the mean ± SD. The Shapiro–Wilk test was used to assess normality, and homoscedasticity was evaluated through visual inspection of residual plots. Because the sample consisted of highly homogeneous elite athletes, no covariates (e.g., age, body mass index, training volume) were included in the statistical models. To compare mean values between performance groups, Student's unpaired *t*‐test was applied when assumptions of normality and homogeneity of variance were met. When equality of variances was violated, Welch's *t*‐test was used. For variables that were non‐normally distributed, the Mann–Whitney *U*‐test was applied. Statistical significance was set at *P* < 0.05. The effect size was calculated using Cohen's *d* coefficient for Student's and Welch's *t*‐tests. For the Mann–Whitney *U*‐test, the effect size was represented by rank biserial correlation. A correlation matrix was computed using Pearson's or Spearman's correlation coefficient, depending on the data distribution. Statistical analyses were performed with JASP v.0.19.1.0 (JASP Team, Amsterdam, The Netherlands), and all figures were generated using Prism v.8 (GraphPad Software Inc., La Jolla, CA, USA).

## 3 RESULTS

### 3.1 General characteristics

Characteristics of male and female athletes are displayed in Table 2. As anticipated, males were significantly taller and heavier than females. Although no significant difference was observed in the body mass index, females displayed a significantly higher quadriceps skinfold thickness in comparison to males. No significant differences were observed between males in Tiers 3–4 and 5.

**TABLE 2:** Main characteristics of athletes.

Characteristics	Female		Male					Sex effect	Δ%	ES
		All males	Tier 3–4	Tier 5	Calibre effect	Δ%	ES			
Number of subjects	10	19	11	8						
Age, years)	22 ± 5	22 ± 4	21 ± 4	23 ± 4	0.333	8.6	−0.27^†^	0.974	−0.2	0.13
Height, cm	170 ± 5	181 ± 6	181 ± 6	181 ± 5±	0.973	0.1	−0.02	**<0.001**	6.4	−2.04
Weight, kg	59.7 ± 4.8	70.7 ± 6.8	72.0 ± 6.9	69.0 ± 6.6	0.442	−4.2	0.44	**<0.001**	18.5	−1.78
BMI, kg m^−2^	20.6 ± 1.1	21.6 ± 1.3	22.0 ± 1.4	21.9 ± 1.0	0.124	−4.4	0.75	0.072	4.5	−0.73
Skinfold_quad_, mm	16.0 ± 4.3	5.3 ± 1.6	5.8 ± 2.0	4.6 ± 0.6	0.147	−20.1	0.80	**<0.001**	−67.2	1.00^†^

*Note*: Values in bold indicate *P* < 0.05 for sex or calibre differences. ES, effect size (Cohen's *d* or ^†^Mann–Whitney) and Δ%, delta (%) are calculated with females as reference for sex comparisons and with Tier 3–4 as reference for calibre comparisons. Abbreviations: BMI, body mass index; Skinfold_quad_, skinfold thickness at the right vastus lateralis.

### 3.2 Graded exercise test

Sex differences between males and females during the graded exercise test are presented in Table 3. At all intensities (VT_1_, VT_2_ and Max), ${\dot{V}}_{E}$, ${\dot{V}}_{O_{2}\max}$ and vV were significantly higher for the males athletes. Moreover, maximal blood lactate concentration was significantly lower for females. Regarding the performance levels, Tiers 3–4 had a significantly higher first threshold (${\dot{V}}_{O_{2}}$%VT_1_), whereas ${\dot{V}}_{O_{2}}$ was higher at the VT_2_ and at Max for the Tier 5 group. The vV was also significantly higher at VT_2_ for the Tier 5 group.

**TABLE 3:** Cardiopulmonary parameters of athletes at the first and second ventilatory thresholds and maximal values.

Incremental exercise test		Female	Male						Sex effect	Δ%	ES
			All males	Tier 3–4	Tier 5	Calibre effect	Δ%	ES			
VT_1_	Number of subjects	10	19	11	8						
	$S_{pO_{2}}$, %	98.7 ± 0.9	98.2 ± 0.7	98.3 ± 0.7	98.2 ± 0.7	0.820	−0.2	0.08^†^	0.267	−0.4	0.49
	${\dot{V}}_{E}$, L min^−1^	61.8 ± 4.5	82.2 ± 10.5	82.9 ± 11.5	81.2 ± 9.5	0.731	−2.2	0.16	**<0.001**	33.0	−2.53
	HR, beats min^−1^	158 ± 11	156 ± 6	159 ± 6	153 ± 6	0.055	−3.6	0.96	0.607	−1.3	0.22
	${\dot{V}}_{O_{2}}$, mL min^−1^ kg^−1^	40.5 ± 3.2	47.8 ± 2.1	47.3 ± 1.5	48.4 ± 2.3	0.312	2.2	−0.48	**<0.001**	17.8	−2.89
	${\dot{V}}_{O_{2}}$, %	67.1 ± 4.3	69.1 ± 3.2	70.4 ± 3.2	67.3 ± 2.2	**0.031**	−4.4	1.10	0.192	3.2	−0.54
	REv, mL kg^−1^ (vertical m)^−1^	2.27 ± 0.12	2.22 ± 0.09	2.21 ± 0.08	2.23 ± 0.10	0.552	1.1	−0.28	0.179	−2.4	0.54
	vV, m h^−1^	1072 ± 84	1292 ± 42	1286 ± 30	1300 ± 57	0.579	1.1	−0.13^†^	**<0.001**	20.5	−1.00^†^
VT_2_	$S_{pO_{2}}$, %	97.3 ± 2.0	97.3 ± 1.5	97.9 ± 0.6	96.5 ± 2.0	0.195	−1.4	0.40^†^	0.523	0.0	0.16^†^
	${\dot{V}}_{E}$, L min^−1^	89.5 ± 9.1	123.6 ± 15.1	120.1 ± 15.0	128.4 ± 14.7	0.243	7.0	−0.56	**<0.001**	38.1	−2.55
	HR, beats min^−1^	180 ± 4	180 ± 2	180 ± 6	181 ± 3	0.798	0.4	−0.12	0.961	−0.1	0.02
	${\dot{V}}_{O_{2}}$, mL min^−1^ kg^−1^	51.7 ± 3.7	60.6 ± 4.0	58.9 ± 2.8	63.0 ± 4.3	**0.022**	7.0	−1.17	**<0.001**	16.6	−3.34
	${\dot{V}}_{O_{2}}$, %	85.6 ± 4.4	87.5 ± 3.7	87.5 ± 3.8	87.6 ± 3.8	0.966	0.1	−0.02	0.243	2.0	−0.48
	REv, mL kg^−1^ (vertical m)^−1^	2.26 ± 0.09	2.22 ± 0.09	2.23 ± 0.11	2.21 ± 0.06	0.675	−0.9	0.20	0.255	−1.8	0.45
	vV, m h^−1^	1379 ± 73	1639 ± 113	1586 ± 65	1712 ± 127	**0.028**	8.0	−1.25	**<0.001**	18.8	−2.56
Max	$S_{pO_{2}}$, %	92.2 ± 2.6	94.1 ± 2.5	95.0 ± 1.8	93.0 ± 3.0	0.140	−2.1	0.85	0.080	2.1	−0.44^†^
	${\dot{V}}_{E}$, L min^−1^	125.5 ± 7.7	181.2 ± 16.0	179.9 ± 18.9	183.0 ± 12.0	0.683	1.8	−0.19	**<0.001**	44.3	−4.42
	HR, beats min^−1^	192 ± 10	193 ± 5	194.3 ± 5.4	191.0 ± 3.3	0.151	−1.7	0.70	0.715	0.7	−0.16
	${\dot{V}}_{O_{2}}$, mL min^−1^ kg^−1^)	60.4 ± 3.3	69.3 ± 4.2	67.4 ± 3.1	72.0 ± 4.4	**0.015**	6.9	−1.25	**<0.001**	14.3	−2.22
	vV, m h^−1^	1740 ± 99	2059 ± 115	2018 ± 66	2116 ± 147	0.144	4.8	−0.85	**<0.001**	18.3	−2.90
	REv, mL kg^−1^ (vertical m)^−1^	2.09 ± 0.08	2.02 ± 0.08	2.00 ± 0.09	2.04 ± 0.06	0.277	2.0	−0.52	**0.030**	−3.5	0.89
	[BLa]_max_, mmol L^−1^	11.2 ± 2.0	12.9 ± 1.9	13.1 ± 1.2	12.7 ± 2.6	0.691	−3.1	0.20	**0.033**	15.0	−0.88

*Note*: Values in bold indicate *P* < 0.05 for sex or calibre differences. ES, effect size (Cohen's *d* or ^†^Mann–Whitney) and Δ%, delta (%) are calculated with females as reference for sex comparisons and with Tier 3–4 as reference for calibre comparisons. Abbreviations: [BLa]_max_, maximal blood lactate concentration; HR, heart rate; Max, maximal intensity; REv, running vertical economy; $S_{pO_{2}}$, pulse oxygen saturation; ${\dot{V}}_{E}$, minute ventilation; ${\dot{V}}_{O_{2}}$, oxygen uptake; vV, vertical velocity; VT_1_, first ventilatory threshold.; VT_2_, second ventilatory threshold.

### 3.3 Circulating blood volumes

Sex differences in haematological data are represented in Table 4. All parameters were significantly higher in males. No significant differences were observed between males in Tiers 3–4 and 5 regarding haematological parameters.

**TABLE 4:** Haematological data of athletes.

Haematological data	Female	Male						Sex effect	Δ%	ES
		All males	Tier 3–4	Tier 5	Calibre effect	Δ%	ES			
Number of subjects	9	18	10	8						
Hbmass, g	657 ± 60	958 ± 141	943 ± 115	977 ± 174	0.624	3.6	−0.24	**<0.001**	45.7	−2.49
HbmassBM, g kg^−1^	11.2 ± 1.0	13.7 ± 1.3	13.4 ± 1.1	14.2 ± 1.5	0.186	6.5	−0.65	**<0.001**	22.7	−2.03
RBCV, mL	2080 ± 189	3036 ± 458	3015 ± 334	3063 ± 603	0.834	1.6	−0.10	**<0.001**	46.0	−2.44
PV, mL	2711 ± 285	3337 ± 314	3316 ± 343	3362 ± 296	0.769	1.4	−0.14	**<0.001**	23.1	−2.05
BV, mL	4791 ± 432	6374 ± 666	6332 ± 569	6426 ± 810	0.778	1.5	−0.14	**<0.001**	33.0	−2.63
Hct, %	43.4 ± 2.0	47.5 ± 3.3	47.6 ± 3.0	47.4 ± 3.8	0.892	−0.5	0.66	**0.003**	9.3	−1.37
[Hb], g dL^−1^	13.7 ± 0.7	15.0 ± 0.9	14.9 ± 1.0	15.1 ± 0.9	0.584	1.7	−0.26	**0.002**	9.0	−1.42

*Note*: Values in bold indicate *P* < 0.05 for sex or calibre differences. ES, effect size (Cohen's *d*) and Δ%, delta (%) are calculated with females as reference for sex comparisons and with Tier 3–4 as reference for calibre comparisons. Abbreviations: BV, blood volume; [Hb], haemoglobin concentration; Hbmass, haemoglobin mass; HbmassBM, relative haemoglobin mass; Hct, haematocrit; PV, plasma volume; RBCV, red blood cell volume.

### 3.4 Correlation matrix

**FIGURE 2:**
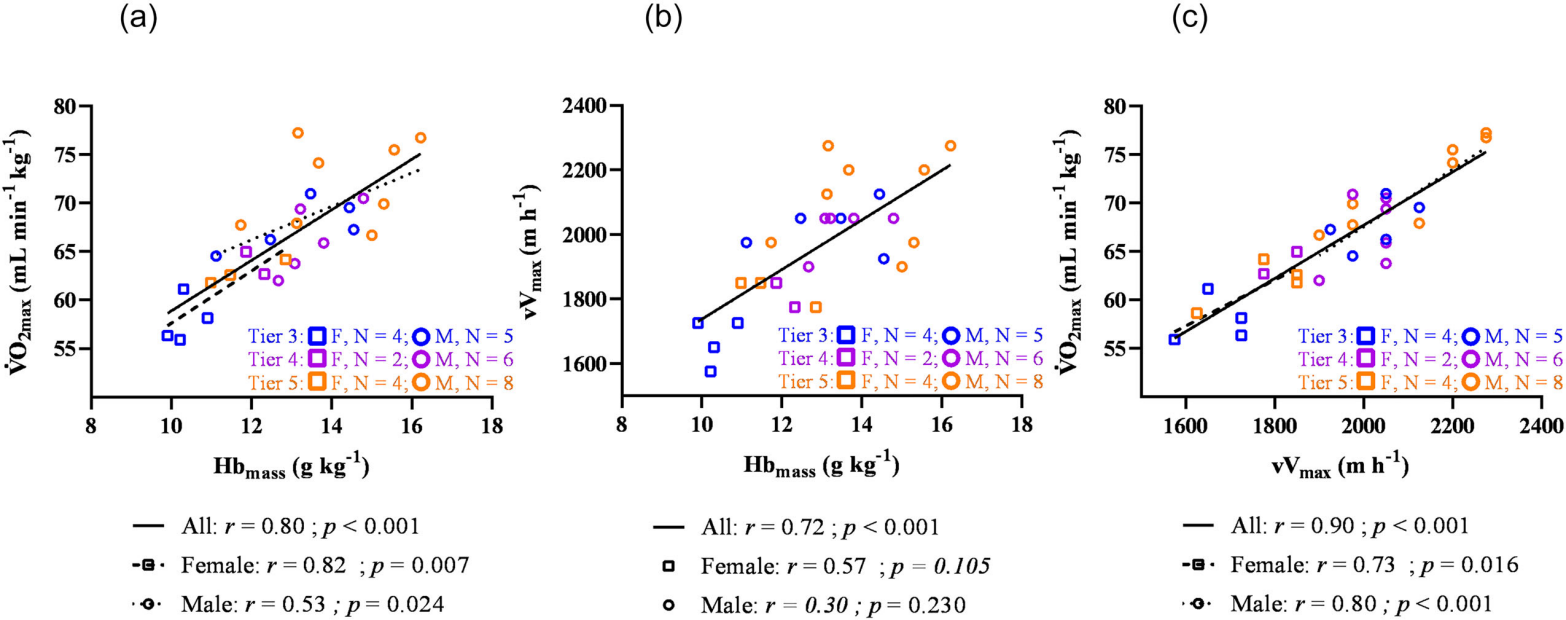
(a) Correlation between relative haemoglobin mass (HbmassBM) and maximal oxygen consumption (${\dot{V}}_{O_{2}\max}$). (b) Correlation between vertical velocity (vV) and relative haemoglobin mass (HbmassBM). (c) Correlation between vertical velocity (vV) and maximal oxygen consumption (${\dot{V}}_{O_{2}\max}$). Females are represented by squares and males by circles. Tier 3 is represented in blue, Tier 4 in purple and Tier 5 in orange.

Correlations between haematological parameters and aerobic performance metrics are shown in Table 5. The HbmassBM was significantly correlated with ${\dot{V}}_{O_{2}\max}$ in the pooled sample (*R* ^2^ = 0.63; *P* < 0.001) and within the female (*R* ^2^ = 0.67; *P* = 0.007) and male (*R* ^2^ = 0.28; *P* = 0.023) subgroups. However, no significant correlation was observed within Tier 3–4 (*R* ^2^ = 0.35; *P *= 0.069) or Tier 5 (*R* ^2^ = 0.12; *P* = 0.405) athletes (Figure 2).

**TABLE 5:** Correlation matrix of physical performance parameters with haematological parameters.

Correlation matrix		Hbmass (g)	HbmassBM (g kg^−1^)	BV (mL)	[Hb] (g dL^−1^)
All	${\dot{V}}_{O_{2}}$__VT2_, mL min^−1^ kg^−1^	0.601*	0.698**	0.677**	0.425
	vV_VT2_, m h^−1^	0.620*	0.651**	0.638**	0.452*
	${\dot{V}}_{O_{2}\max}$, mL min^−1^ kg^−1^	0.671**	0.796**	0.677**	0.521*
	vV_max_, m h^−1^	0.693**	0.721**	0.706**	0.531*
Female	${\dot{V}}_{O_{2}}$__VT2_, mL min^−1^ kg^−1^	0.567	0.517	0.550	−0.033
	vV_VT2_, m h^−1^	0.689*	0.474	0.604	0.120
	${\dot{V}}_{O_{2}\max}$, mL min^−1^ kg^−1^	0.669*	0.819*	0.340	0.607
	vV_max_, m h^−1^	0.739*	0.575	0.359	0.494
All males	${\dot{V}}_{O_{2}}$__VT2_, mL min^−1^ kg^−1^	0.015	0.309	0.053	−0.118
Tier 3–4		0.142	0.323	0.490	−0.349
Tier 5		−0.229	0.079	−0.276	−0.104
All males	vV_VT2_, m h^−1^	−0.019	0.186	−0.035	<0.001
Tier 3–4		0.181	0.002	0.214	0.037
Tier 5		−0.234	0.002	−0.242	−0.208
All males	${\dot{V}}_{O_{2}\max}$, mL min^−1^ kg^−1^	0.221	0.530*	0.255	0.087
Tier 3–4		0.396	0.595	0.554	0.005
Tier 5		0.059	0.343	0.080	0.013
All males	vV_max_, m h^−1^	0.094	0.298	0.110	0.035
Tier 3–4		0.537	0.298	0.581	0.202
Tier 5		−0.144	0.150	−0.115	−0.177

*Note*: Values are Pearson or Spearman correlation coefficients. Correlation matrix: *P < 0.05 and **P < 0.001. Abbreviations: BV, blood volume; [Hb], haemoglobin concentration; Hbmass, haemoglobin mass; HbmassBM, relative haemoglobin mass; Hct, haematocrit; Max, maximal effort; PV, plasma volume; RBCV, red blood cell volume; ${\dot{V}}_{O_{2}}$, oxygen uptake; VT_2_, second ventilatory threshold; vV, vertical velocity.

## 4 DISCUSSION

The main findings of the present study were as follows: (1) according to previous research, we observed a significant correlation between HbmassBM and ${\dot{V}}_{O_{2}\max}$ in the pooled group (All), in females and in males; (2) among all haematological parameters assessed, only HbmassBM was significantly correlated with ${\dot{V}}_{O_{2}\max}$ in both females and males; (3) HbmassBM values in elite SkiMo athletes (both females and males) appear to be consistent with those reported in other endurance summer or winter sports; and (4) Tier 5 male athletes showed higher ${\dot{V}}_{O_{2}}$ values at VT_2_ and at maximal intensity compared with Tier 3–4 athletes.

### 4.1 Relationship between Hbmass and aerobic parameters

Although the relationship between Hbmass and ${\dot{V}}_{O_{2}\max}$ has been well documented across various endurance sports, the novelty of the present study lies in the investigated elite population in a sport that has unique physiological demands: SkiMo involves prolonged uphill locomotion at altitude, with a substantial upper‐body contribution. This distinguishes it from many other endurance activities (e.g., cycling, running) and justifies a dedicated analysis to test our hypothesis that ski mountaineers would have higher Hbmass values. Our findings provide additional evidence supporting the robust association between HbmassBM and ${\dot{V}}_{O_{2}\max}$ (Figure 2), extending observations in other endurance sports.

Overall, there is a strong correlation (*r *= 0.8) between HbmassBM and ${\dot{V}}_{O_{2}\max}$ for the pooled group. These results align with previous findings across various endurance sports. Goodrich et al. (2018) reported a strong positive correlation between HbmassBM and ${\dot{V}}_{O_{2}\max}$ (*r *= 0.85, *P* < 0.001) in 33 endurance‐trained cyclists and triathletes. In the present study, it is of interest that correlations were significant in both males and females (Figure 2). Likewise, Gore et al. (1997) found significant correlations between relative HbmassBM and ${\dot{V}}_{O_{2}\max}$ across three groups: female rowers (*n* = 17) showed the strongest correlation (*r* = 0.92, *P* < 0.001), male rowers (*n* = 12) had a moderate correlation (*r *= 0.79, *P* < 0.005), and male runners (*n* = 33) exhibited the weakest correlation (*r *= 0.48, *P* = 0.005). These consistent associations across various endurance sports highlight the crucial role of HbmassBM in aerobic capacity. The absence of significant correlations between HbmassBM and ${\dot{V}}_{O_{2}\max}$ within Tier 3–4 and Tier 5 subgroups is likely to be explained by the restricted range and small sample size within each performance tier, which limit statistical power despite exhibiting similar correlation coefficients.

Regarding other haematological parameters, BV and [Hb] were also significantly correlated with ${\dot{V}}_{O_{2}\max}$, but only when males and females were pooled together. This is likely to reflect sex‐related heterogeneity in absolute values of [Hb] and BV, because males typically have higher haematological values and ${\dot{V}}_{O_{2}\max}$. However, this relationship did not hold within each sex, where individual variability is smaller. Moreover, [Hb] was not significantly correlated with ${\dot{V}}_{O_{2}\max}$ or vV. This might be attributable to the fact that [Hb] can be influenced by the menstrual cycle or by acute fluctuations in PV, which are affected by factors such as training status, hydration or environmental exposure (Heinicke et al., 2003; Jimenez et al., 1999; Krumm & Faiss, 2021). Importantly, the absence of differences in Hct and [Hb] between performance tiers, despite clear differences in Hbmass and BV, is likely to reflect greater plasma volume expansion in the higher‐performing athletes. This phenomenon, often described as dilutional ‘pseudoanaemia’, is well documented in endurance populations (Shaskey & Green, 2000) and underscores why [Hb] and Hct are less informative in athletes compared with absolute measures of Hbmass (Malczewska‐Lenczowska et al., 2013). In contrast, Hbmass directly represents the total haemoglobin content in the body and is not affected by changes in PV. Thus, Hbmass appears to be a more robust and stable indicator of the oxygen‐carrying capacity in endurance athletes compared with [Hb], making it particularly relevant for evaluating aerobic performance potential and monitoring training adaptation (Kasiak et al., 2025). Our findings therefore reinforce the physiological and methodological importance of CO‐rebreathing‐derived Hbmass assessment, especially in sports such as SkiMo, where plasma volume can fluctuate substantially across training periods and environmental exposures.

In addition to the haematological differences discussed above, we observed that peak blood lactate concentration was significantly lower in females compared with males (*P* = 0.033). This difference might be attributed, in part, to sex‐related physiological characteristics. Males generally have greater muscle mass and a higher proportion of type II fibres (Joyner et al., 2025), which are more glycolytic during high‐intensity exercise (Edvardsen et al., 2014). These factors are likely to contribute to the higher peak lactate values observed in male athletes.

### 4.2 HbmassBM values

Owing to the unique demands of their sport, elite ski mountaineers are considered the endurance athletes with the highest exposure to hypoxic conditions (i.e., total time spent in a low‐oxygen environment) across all disciplines (Faiss et al., 2014). Therefore, we hypothesized that elite ski mountaineers might gain physiological benefits from this prolonged hypoxic exposure and would have higher Hbmass values in comparison to other endurance sports performed near sea level. However, our results do not confirm this hypothesis. Despite training predominantly at altitudes above 1500–2000 m a.s.l., SkiMo athletes display similar HbmassBM values to those reported by Oberholzer et al. (2024) in a large cohort of various endurance athletes (21 women and 86 men). Hence, the mean HbmassBM value for females (11.2 g kg^−1^) seems to be slightly lower than values reported in other elite athletes; e.g., 12.3 g kg^−1^ for female elite cyclists (Garvican et al., 2010); 12.0 g kg^−1^ in world‐class middle‐ and long‐distance runners and racewalkers (Heikura et al., 2018); and 13.0 g kg^−1^ in elite biathletes (Heinicke et al., 2004). In the present study, the mean value for males was 13.7 g kg^−1^, and in Tier 5 athletes 14.2 g kg^−1^, which is comparable to other previously reported values of 14.0 g kg^−1^ in other endurance sports (Heikura et al., 2018; Heinicke et al., 2004, 2001; Mancera‐Soto et al., 2025; Wehrlin & Marti, 2006; Wehrlin & Steiner, 2021). Overall, Hbmass remains relatively stable, with a within‐subject coefficient of variation of ∼3%–4% throughout the season (Eastwood et al., 2012), probably owing to fluctuations in training load (Garvican et al., 2010). Moreover, no significant changes were reported in Hbmass over the course of 1 year in trained cyclists, further reinforcing the notion that Hbmass is a relatively stable haematological marker in well‐trained endurance athletes (Astolfi et al., 2021). In the present study, measurements were conducted in June, shortly after the off‐season break, and no altitude training camps were performed before the measurements. One may therefore speculate that the Hbmass values were not yet optimized in these athletes.

It is important to note that absolute Hbmass is strongly influenced by lean body mass (Falz et al., 2019), meaning that HbmassBM can vary across different sports, particularly when body fat percentage differs between disciplines. From a physiological perspective, differentiating absolute and relative Hbmass (HbmassBM) is important when assessing endurance performance (Oberholzer et al., 2024). The absolute Hbmass reflects the total oxygen‐carrying capacity, whereas HbmassBM better represents oxygen‐transport efficiency relative to body size. In weight‐bearing sports involving substantial vertical displacement, such as SkiMo, a higher HbmassBM might be particularly relevant for explaining performance differences, even when absolute Hbmass values are similar. We did not measure lean body mass, only skinfold on vastus lateralis. However, SkiMo involves steep uphill climbs, where body fat percentage is closely linked to performance (Fornasiero et al., 2017). Given the high aerobic demands of this sport, these athletes are likely to have a very low body fat percentage, as shown previously, with average body fat percentages of 15.2% in females and 6.6% in males (Schorderet et al., 2025) or 9.8% in nine elite SkiMo athletes (seven men and two women) (Fornasiero et al., 2017). The high‐performance level of our sample suggests similar or lower fatness. Therefore, our HbmassBM values should be interpreted in the context of other elite endurance sports, where athletes typically exhibit low body fat percentages, enabling more meaningful comparisons.

### 4.3 Tier3–4 versus Tier5

**FIGURE 3:**
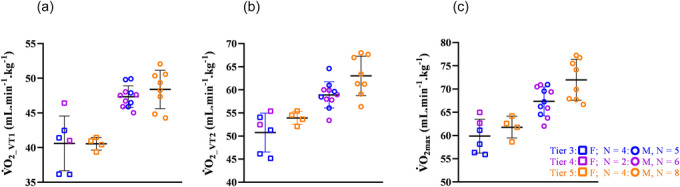
(a) Comparison of oxygen consumption at the first ventilatory threshold (${\dot{V}}_{O_{2}}$__VT1_) between Tiers 3–4 and Tier 5. (b) Comparison of oxygen consumption at the second ventilatory threshold (${\dot{V}}_{O_{2}}$__VT2_) between Tiers 3–4 and Tier 5. (c) Comparison of maximal oxygen consumption (${\dot{V}}_{O_{2}\max}$) between Tiers 3–4 and Tier 5. Females are represented by squares and males by circles. Tier 3 is represented in blue, Tier 4 in purple and Tier 5 in orange.

It is well established that elite athletes possess distinct physiological characteristics that cannot be inferred from subelite data (Burke et al., 2024). For this reason, we analysed the differences between Tier 3–4 and Tier 5. Consistent with our hypothesis, Tier 5 displayed slightly higher ${\dot{V}}_{O_{2}\max}$ values than Tier 3–4 athletes (Figure 3). In line with a previous SkiMo study (Lasshofer et al., 2021), we also observed significant differences in ${\dot{V}}_{O_{2}}$ at both VT_2_ and maximal levels between the two groups, supporting our third hypothesis. Lasshofer et al. (2021) reported ${\dot{V}}_{O_{2}\max}$ values of 65.6 and 71.2 mL min^−1^ kg^−1^ for sub‐elite and elite groups, respectively; values comparable to those found in our study (67.4 and 72.0 mL min^−1^ kg^−1^ for Tier 3–4 and Tier 5, respectively).

Given that no significant differences were found in haematological parameters between Tier 3–4 and Tier 5, Tier 5 athletes might possess other favourable adaptations that explain their higher ${\dot{V}}_{O_{2}\max}$. This might be convective (e.g. cardiac output, stroke volume) and diffusive (pulmonary or muscle) components of ${\dot{V}}_{O_{2}\max}$ (Rønnestad et al., 2019; Wagner, 2000). Additional ski‐specific factors, such as upper‐limb strength and efficiency, not assessed in the present study, also warrant further investigation. Tier 3–4 athletes exhibited a higher relative oxygen uptake at VT_1_ (${\dot{V}}_{O_{2}}$%VT_1_) than Tier 5. This difference is unlikely to reflect greater efficiency, because REv did not differ between groups. One may speculate that these differences are related to different ventilatory control in exercise conditions between the groups, but without any recorded data for confirmation.

Overall, higher ${\dot{V}}_{O_{2}\max}$ values have generally been reported in other winter endurance sports, particularly in Nordic ski: 73.5 to 84.3 mL min^−1^ kg^−1^ for Olympic medalists and non‐medalists in Nordic skiing disciplines (cross‐country, biathlon and Nordic combined) (Tønnessen et al., 2015). In SkiMo athletes, previous studies have reported ${\dot{V}}_{O_{2}\max}$ values comparable to or slightly lower than those observed in the present cohort, typically ranging from ∼67 to 80 mL min^−1^ kg^−1^ (Duc et al., 2011; Fornasiero et al., 2017; Lasshofer et al., 2021). Therefore, our findings align well with existing literature, suggesting that elite SkiMo athletes exhibit aerobic capacities similar to those of other high‐level endurance athletes. These values are slightly higher than those observed in our Tier 5 athletes, probably reflecting both the greater competitive density in Nordic skiing, a long‐established Olympic sport, and the highly glycolytic nature of the SkiMo Olympic events (i.e., sprint and mixed relay). First, with the recent inclusion of SkiMo in the Olympic programme, it might be argued that the competitive international density will be likely to increase, potentially driving a rise in both performance levels and physiological markers, such as ${\dot{V}}_{O_{2}\max}$, in the coming years. Second, the majority of the SkiMo athletes in our cohort specialized in sprint or mixed relay events. Sprint races involve three or four efforts of ∼3 min each, with ∼20 min of recovery between efforts, whereas mixed relay events consist of two laps of ∼10–15 min per athlete (Fornasiero et al., 2023, 2025). In contrast, only a few athletes in our cohort focus primarily on vertical races (a single ascent lasting 20–30 min) or individual races, which typically last 75–90 min (Bortolan et al., 2021).

Recent findings indicate that speed at ${\dot{V}}_{O_{2}\max}$ is the best predictor of performance in mixed relay events, whereas sprint performance is most strongly associated with supramaximal speed (Fornasiero et al., 2025). For longer‐duration efforts, such as vertical races, ${\dot{V}}_{O_{2}}$ at VT_2_ appears to be one of the best performance predictors (Fornasiero et al., 2017; Lasshofer et al., 2021).

To date, no study has directly compared the aerobic profiles of SkiMo sprint and distance specialists. However, similar differences have been reported in cross‐country skiing, where sprint athletes typically demonstrate lower ${\dot{V}}_{O_{2}\max}$ values than distance skiers, probably owing to greater anaerobic demands and increased muscle mass (Losnegard & Hallén, 2014; Tønnessen et al., 2015). Future studies are warranted to explore these intra‐sport differences and to improve physiological profiling and training strategies for elite SkiMo athletes.

Regarding seasonal variation, longitudinal data on ${\dot{V}}_{O_{2}\max}$ and ventilatory thresholds in SkiMo athletes are currently lacking. Measurements in the present study were conducted during the early preparation phase, well before the competitive season, which might explain, in part, the slightly lower values observed. Future research should examine how cardiorespiratory and haematological variables fluctuate throughout the season and in response to cumulative hypoxic exposure.

One key question in elite sports is: what sets medal‐winning athletes apart when physiological profiles, such as ${\dot{V}}_{O_{2}\max}$, are similar? In our study, Tier 5 athletes had slightly higher ${\dot{V}}_{O_{2}\max}$, but other factors probably contributed. As argued by Joyner and Coyle (2008), elite performance resists reductionist explanations. Consequently, other factors (such as running economy, race strategies and anaerobic capacity) might play a crucial role in differentiating performance among top‐tier athletes. Exercise economy has been shown to vary considerably among trained individuals (Lucia et al., 2006; Saunders et al., 2004). Given that running economy can differ by 15%–20% among athletes with similar ${\dot{V}}_{O_{2}\max}$ values (Kearney & Van Handel, 1989), it becomes a key determinant of performance (Joyner & Coyle, 2008; McLaughlin et al., 2009). Moreover, athletes who are more economical in skiing also tend to demonstrate a high running economy, which could be linked to other intrinsic factors (Losnegard et al., 2014). Given that running economy is also trainable (Millet & Bentley, 2004), optimizing this parameter through targeted interventions could provide a competitive edge when ${\dot{V}}_{O_{2}\max}$ alone is no longer the primary differentiating factor. Additionally, a recent study (Fornasiero et al., 2017) reported that gross efficiency (GE) was strongly correlated with vertical SkiMo performance and was also linked to the age of the athlete, suggesting a potential effect of experience. Building on these findings, one might expect Tier 5 athletes to exhibit better running economy than Tier 3–4 athletes. However, in our study, vertical running economy (REv) did not differ significantly between these groups at VT_2_. Future studies might benefit from assessing REv over longer exercise durations, because our calculations were based on the average ${\dot{V}}_{O_{2}}$ values from the final 20 s preceding VT_1_, VT_2_ and maximal effort.

Future relevant applied research might focus on the different forms of hypoxic exposure (such as live‐high train‐high, live‐high train‐low, or repeated intermittent hypoxic sessions) and assess their effect on Hbmass in elite ski mountaineers. Identifying the optimal ‘hypoxic dose’, including altitude level, duration and frequency of exposure, would help to clarify which strategies are most effective for this population. Moreover, it remains unknown how individual haematological profiles influence recovery, susceptibility to fatigue and tolerance to high training volumes in SkiMo athletes. Addressing these questions would provide valuable insights for optimizing training periodization and altitude‐based interventions in this unique endurance discipline.

### 4.4 Strengths and limitations

A key strength of this study lies in the exceptional performance level of the participants. We had the opportunity to work with a large cohort of elite athletes, allowing us (much like our Norwegian and Swedish counterparts in Nordic skiing research) to explore the physiological specificities of SkiMo at the highest level. The robustness of our data is supported by the significant correlations observed between Hbmass and ${\dot{V}}_{O_{2}\max}$ (up to *r* = 0.82), both within sexes and in the pooled group.

The present study presents also some limitations. First, it is well known that several confounding factors might influence haematological measurements. Preceding acute exercise, body position and hormonal fluctuations (e.g., menstrual cycle phase) are known to affect blood variables (Krumm & Faiss, 2021). Although the Hbmass measurements were performed ∼40 min after the end of the incremental test, minimizing the likely impact of exercise‐induced haemoconcentration or splenic contraction, a residual effect cannot be excluded fully. Nonetheless, the reported Hbmass values closely align with those reported in other elite athletes (Oberholzer et al., 2024), lending credibility to our data.

Second, the assessment of body composition was limited. Estimations of sex differences in fatness were based solely on the vastus lateralis skinfold, without full‐body assessment, such as dual‐X‐ray absorptiometry (DXA). Consequently, we were unable to normalize Hbmass to fat‐free mass; a key methodological consideration in sex‐difference research to reduce confounding (Goodrich et al., 2020; Tripp et al., 2025). Likewise, body mass index offers limited interpretative value in elite athletes, because it does not distinguish between lean and fat mass, which can influence physiological interpretation. However, even without this adjustment, significant sex‐specific and pooled correlations between Hbmass and ${\dot{V}}_{O_{2}\max}$ suggest that the observed relationships remain strong.

Third, the sex distribution was unbalanced (19 males vs. 10 females), limiting statistical power for between‐sex comparisons. Specifically, the small female sample size prevented us from conducting two‐way ANOVAs to analyse interactions between sex and performance tiers. This restricts the depth of our comparative analyses.

Fourth, we did not assess iron status or related haematological markers, which limits our ability to evaluate their potential contribution to sex differences in Hbmass. Including markers such as ferritin, transferrin saturation or hepcidin would provide important contextual insights.

Fifth, although the menstrual cycle phase was recorded, it was not controlled for in the testing schedule. This might introduce variability in haematological outcomes; however, accumulating evidence indicates minimal impact of menstrual phase on performance and oxygen‐carrying capacity in both normoxic and hypoxic conditions (Citherlet et al., 2024; Elliott‐Sale et al., 2020)

Sixth, data collection occurred during the early pre‐season period. Given that athletes were not yet in peak competitive condition, haematological and performance values might differ slightly from true in‐season peak values. This timing might therefore limit generalization to later phases of the competitive season.

Seventh, although the typical hypoxic exposure and training habits of athletes are described qualitatively, we did not quantify individual training load, hypoxic dose or recent altitude exposure. These unmeasured confounders might contribute to variability in Hbmass, lactate responses and performance measures.

Lastly, although our study focused on Hbmass and ${\dot{V}}_{O_{2}\max}$, a more comprehensive understanding of endurance performance would benefit from investigating additional physiological determinants, such as cardiac output, mitochondrial density or muscle oxidative capacity. Although non‐invasive techniques exist, implementing them in elite athlete populations poses ethical, logistical and technical challenges.

Together, these limitations reflect the uniqueness and complexity of conducting physiological profiling in elite athletes, particularly of Tier 5.

## 5 CONCLUSION

Our main findings are as follows: (i) HbmassBM was correlated with ${\dot{V}}_{O_{2}\max}$ in the pooled group of female and male athletes; (ii) among haematological parameters, only HbmassBM was correlated with ${\dot{V}}_{O_{2}\max}$ in both sexes; and (iii) significant differences in ${\dot{V}}_{O_{2}}$ at VT_2_ and maximal intensity were found between Tier 3–4 and Tier 5 male athletes.

## AUTHOR CONTRIBUTIONS

Grégoire P. Millet conceptualized the protocol. Forrest Schorderet, Bastien Krumm, Basile Moreillon, Justin Mottet, Antoine Raberin and Nicolas Bourdillon performed data acquisition, treatment and analysis. Forrest Schorderet was responsible for the first draft of the manuscript. Forrest Schorderet, Bastien Krumm, Antoine Raberin, Raphael Faiss, Nicolas Bourdillon and Grégoire P. Millet interpreted the data. All authors approved the final version of the manuscript and agree to be accountable for all aspects of the work in ensuring that questions related to the accuracy or integrity of any part of the work are appropriately investigated and resolved. All people designated as authors qualify for authorship, and all those who qualify for authorship are listed.

## CONFLICT OF INTEREST

All authors declare that they have no conflicts of interest. The results of the study are presented clearly, honestly and without fabrication, falsification or inappropriate data manipulation. The results of the present study do not constitute endorsement by the American College of Sports Medicine.

## FUNDING INFORMATION

None.

## Data Availability

The data that support the findings of this study are available from the corresponding author on reasonable request.
